# Hephaestin and ceruloplasmin facilitate iron metabolism in the mouse kidney

**DOI:** 10.1038/srep39470

**Published:** 2016-12-19

**Authors:** Bo Jiang, Guohao Liu, Jiashuo Zheng, Mengxia Chen, Zaitunamu Maimaitiming, Min Chen, Shunli Liu, Ruiwei Jiang, Brie K. Fuqua, Joshua L. Dunaief, Chris D. Vulpe, Gregory J. Anderson, Hongwei Wang, Huijun Chen

**Affiliations:** 1 Jiangsu Key Laboratory of Molecular Medicine, Medical School of Nanjing University, China; 2 Center for Environmental and Human Toxicology, Department of Physiological Sciences, University of Florida, Gainesville, FL, USA; 3 FM Kirby Center for Molecular Ophthalmology, Scheie Eye Institute, University of Pennsylvania, PA, USA; 4 QIMR Berghofer Medical Research Institute, Brisbane, Queensland, Australia

## Abstract

Multicopper ferroxidases (MCFs) play an important role in cellular iron homeostasis. However, the role of MCFs in renal metabolism remains unclear. We used Hephaestin (*Heph*) and Ceruloplasmin (*Cp*) single or double (*Heph*/*Cp*) knockout (KO) mice to study the roles of MCFs in the kidney. Renal iron levels and the expression of iron metabolism genes were examined. The non-heme iron content both in the renal cortex and medulla of *Heph*/*Cp* KO mice was significantly increased. Perls’ Prussian blue staining showed iron accumulation on the apical side of renal tubular cells in *Heph*/*Cp* KO mice. A significant increase in ferritin protein expression was also observed in the renal medulla and cortex of *Heph*/*Cp* KO mice. Both DMT1 and TfR1 protein expression were significantly decreased in the renal medulla of *Heph*/*Cp* KO mice, while the expression of DMT1 protein was significantly increased in the renal cortex of these animals. Significant increase in proteinuria and total urinary iron was observed in the double knockout mice, and this was associated with compromised structural integrity. These results suggest that KO of both the HEPH and CP genes leads to kidney iron deposition and toxicity, MCFs could protect kidney against a damage from iron excess.

Multicopper ferroxidases (MCFs) are known to play a central role in iron nutrition and homeostasis in organisms ranging from yeast to humans^1^. The vertebrate MCFs, Ceruloplasmin (CP), Hephaestin (HEPH), and Zyklopen (ZP), are hypothesized to facilitate iron transport in diverse tissues by oxidizing ferrous iron to the ferric form so that it can bind to the circulating ferric iron carrier transferrin (TF). In these reactions, electrons are transferred from ferrous iron to the type I copper sites of MCFs. The electrons are then transferred to the MCF type II/type III copper site, where molecular oxygen is reduced to water^2^. Our previous work demonstrated that HEPH is located in a supra-nuclear compartment and on the basolateral membrane of intestinal enterocytes^3^. Apical iron administration can lead to the mobilization of HEPH from intracellular sites to the basolateral membrane^4^. HEPH is most strongly expressed in the small intestine, but it has also been found to be expressed in the kidney^5^. CP is mainly found as a soluble serum protein originating from the liver, but it is also found as a glycosylphosphatidylinositol (GPI)-linked protein in astrocytes and the kidney^6^. Mutations in the Cp gene lead to iron accumulation in multiple tissues in humans^7^ and mice^8^.

The role of MCFs and a range of other proteins involved in iron metabolism in iron transport in the kidney remains poorly understood^9^,^10^,^11^. In general, over 99% of the iron filtered by the glomeruli is reabsorbed^9^. Wareing *et al*. suggested that iron was reabsorbed by late proximal tubule segments and early distal tubule segments^12^. Plasma TF (Mr 78,000) can pass through the glomerulus filter^13^,^14^ and be reabsorbed in the proximal tubule via transferrin receptor 1 (TFR1). TFR1 is highly expressed in the proximal tubules of the renal cortex, and is also found on the apical membrane of collecting tubules and distal tubules in the medulla^10^. The TF-TFR1 complex is internalized into endosomes via cubilin-mediated endocytosis, and can be targeted to lysosomes for degradation^15^. Divalent metal-ion transporter 1 (DMT1) is localized to the endosomes and lysosomes within proximal tubule cells, organelles associated with the processing of apically sequestered TF^16^. It is predicted that iron is released from TF at these sites and exported into the cytosol by DMT1^16^. These findings suggest that some TF normally enters the glomerular filtrate, but it is retrieved by specific receptor-mediated uptake in the kidney tubular system. Iron released into the cytoplasm of renal tubular cells is subsequently exported across the basolateral membrane via ferroportin1 (FPN1)^17^. We hypothesize that this is the step which is likely to involve the MCFs as HEPH in the gut and CP in other tissues have been shown to increase the efficiency of FPN1-mediated iron transport^8^,^18^,^19^. DMT1 is also found on the apical membrane of distal tubules, where it can resorb iron from the tubular fluid.

Iron accumulation in the kidney is found in a number of diseases, including Fanconi syndrome, Dent’s disease and chronic renal disease^14^,^20^,^21^. High kidney iron is generally considered to be deleterious to renal function^20^,^21^,^22^ due to its propensity to catalyze the formation of reactive oxygen species^23^, which have the capacity to cause cellular injury. Recent clinical studies indicate that chelation of iron in the kidney has beneficial effects on the course of chronic kidney disease^24^.

In this study we have used *Cp* and *Heph* single, and *Cp*/*Heph* double knockout mice to examine the role of these MCFs in the kidney. These studies showed that knockout of both the *Heph* and *Cp* genes, but not either alone, leads to kidney iron deposition and toxicity. These findings suggest that MCFs may play an important role of protecting kidney against damage from iron excess and that either oxidase is able to compensate for the loss of the other.

## Results

### Iron status at 6 months of age

To evaluate the iron status of the *Heph*/*Cp* KO mice, hematological data were obtained from mice at six months of age. *Heph*/*Cp* KO and *Heph* KO mice, but not *Cp* KO, were anemic based on reductions in hemoglobin (Hb), mean cell volume (MCV), red cell number and hematocrit (Table 1). *Heph*/*Cp* KO mice were significantly more anemic than *Heph* KO animals. Iron levels were significantly lower in the plasma of *Heph*/*Cp* KO, *Cp* KO, and *Heph* KO mice compared to WT control mice, while the hepatic iron concentration was significantly higher in *Heph*/*Cp* KO and *Cp* KO mice compared to WT control and *Heph* KO mice (Table 2). In the kidney, the iron concentration was only significantly increased in *Heph*/*Cp* KO mice compared to the other groups, and not in either of the single gene KOs (Table 2). We then examined the non-heme iron content in the renal cortex and medulla, and found that non-heme iron levels were significantly higher in both the renal cortex and medulla of *Heph*/*Cp* KO mice compared to the other genotypes examined (Table 2).

### Iron distribution and ferritin expression in the renal medulla and cortex

**Figure 1:**
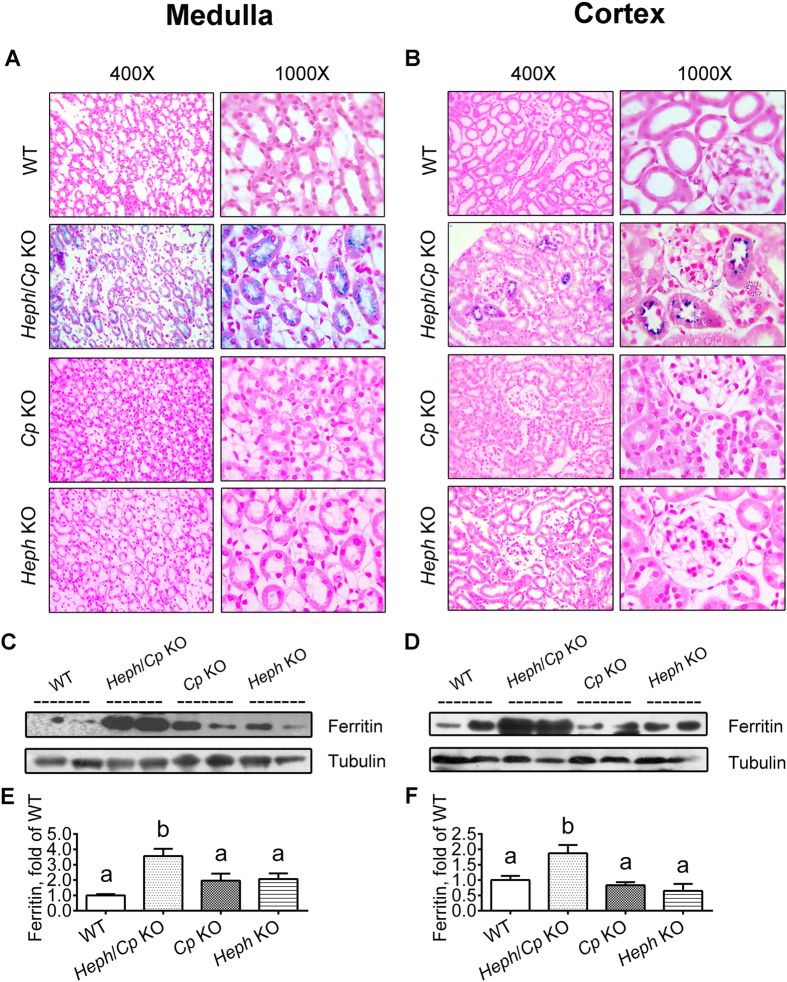
Iron staining and ferritin protein expression in the renal medulla and cortex. Iron levels and distribution were determined by Perls’ Prussian blue staining of kidney sections. Representative photomicrographs of the medulla (**A**) and cortex (**B**) are shown. Strongest iron deposition was observed in the medulla of *Heph*/*Cp* KO mice. Ferritin protein expression was assessed by Western blotting in the renal medulla (**C**) and renal cortex (**D**) of WT, *Heph*/*Cp* KO, *Cp* KO, and *Heph* KO mice. Tubulin served as a loading control. Levels of ferritin protein were quantified by densitometry and these results are shown in parts E (medulla) and F (cortex). Ferritin expression was normalized to tubulin, and then presented as a ratio of ferritin expression in WT mice. Values are presented as the mean ± SEM, *n* = 4–5 mice per genotype. Within each panel, bars without common letters are significantly different, *P* < 0.05.

Iron accumulation was observed by Perls’ Prussian blue staining in the renal medulla of *Heph*/*Cp* KO mice, but no iron was observed in the corresponding region in the kidneys of WT, *Cp* KO or *Heph* KO mice (Fig. 1A). Some iron accumulation was also observed in the renal cortex of *Heph*/*Cp* KO mice, but not in mice with other genotypes (Fig. 1B). At higher magnification (1000X, Fig. 1A,B right hand panels), iron was observed intracellularly, but on the apical side of renal tubular cells and the Loop of Henle in *Heph*/*Cp* KO mice. Consistent with this elevated iron, western blotting showed that ferritin protein expression was increased in the renal medulla (Fig. 1C,E) and cortex (Fig. 1D,F) of *Heph*/*Cp* KO mice, but not in mice of any of the other genotypes All levels were normalized to tubulin and then normalized to ferritin expression in WT mice.

### Expression of iron transport proteins in the renal medulla and cortex

**Figure 2:**
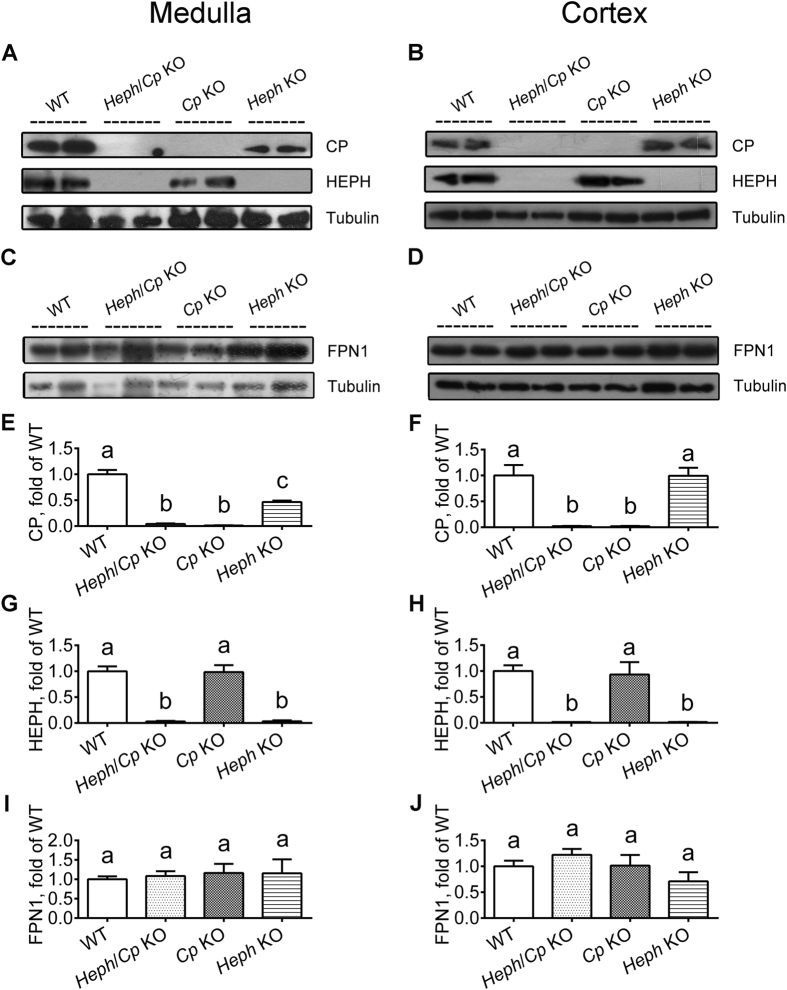
CP, HEPH, and FPN1 protein expression in the renal medulla and cortex. Western blotting was used to investigate CP (**A,B,E,F**), HEPH (**A**,**B**,**G**,**H**) and FPN1 (**C**,**D**,**I**,**J**) expression in the renal medulla (left hand panels) and cortex (right hand panels) of WT, *Heph*/*Cp* KO, *Cp* KO and *Heph* KO mice. Tubulin served as a loading control. For the blots of FPN1, the samples were not heated prior to loading onto the gels. Levels of Cp (**E**,**F**), Heph (**G**,**H**) and FPN1 (**I**,**J**) protein were quantified by densitometry. All levels were normalized to tubulin. Values are presented as the mean ± SEM, *n* = 4–5 mice per group. Within each panel, bars without common letters are significantly different, *P* < 0.05.

**Figure 3:**
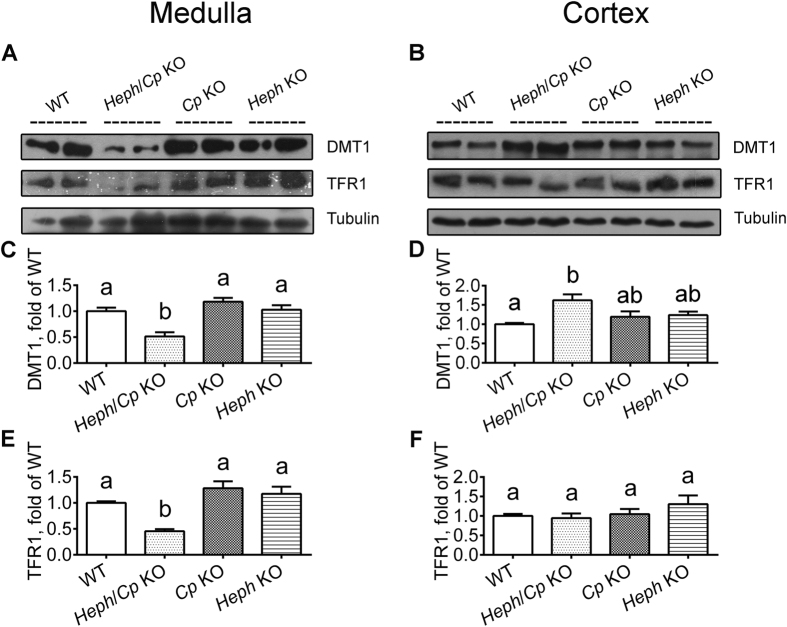
DMT1 (IRE) and TFR1 protein expression in the renal medulla and cortex. Western blotting was used to assess the levels of DMT1 (**A**–**D**) and TFR1 (**A**,**B**,**E**,**F**) protein expression in the renal medulla (left hand panels) and cortex (right hand panels) of WT, *Heph*/*Cp* KO, *Cp* KO, and *Heph* KO mice. Tubulin served as a loading control. For DMT1 blots, 150 μg of protein was loaded per well for renal medulla and 40 μg of protein was loaded per well for renal cortex. Densitometric analysis of the western blots is shown for DMT1 (**C**,**D**) and TFR1 (**E**,**F**) protein. All levels were normalized to tubulin. Values are presented as mean ± SEM, *n* = 4–5 mice per group. Within each panel, bars without common letters are significantly different, *P* < 0.05.

Both HEPH and CP protein were detected in the renal medulla and cortex of WT mice (Fig. 2A,B). As expected, no CP protein expression was detected in the kidneys of *Heph*/*Cp* KO or *Cp* KO mice, and no HEPH protein expression in *Heph*/*Cp* KO or *Heph* KO mouse kidneys. Interestingly, CP protein expression was lower in the renal medulla of *Heph* KO mice than WT, but no difference was detected in the cortex (Fig. 2E,F). FPN1 protein expression was similar for all genotypes in both the cortex and medulla (Fig. 2C,D,I,J). Both DMT1 And TFR1 protein expression were significantly decreased in the renal medulla of *Heph*/*Cp* KO mice compared to the other groups (Fig. 3A,C,E). While DMT1 protein expression was no significantly difference in the renal cortex of genotype mice (Fig. 3B,D). TFR1 protein expression in the renal cortex was similar in mice of all genotypes (Fig. 3B,F).

### Expression of iron transporter genes in the renal medulla and cortex

**Figure 4:**
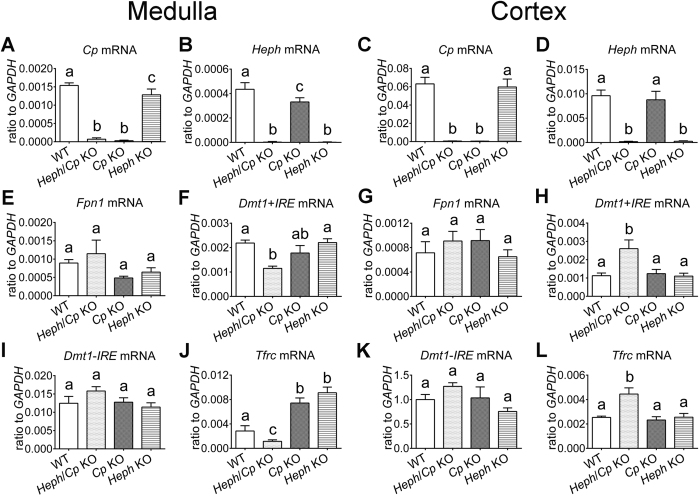
Expression of iron transport-related genes in the renal medulla and cortex. The mRNAs encoding *Cp* (**A**,**C**), *Heph* (**B**,**D**), *Fpn1* (**E**,**G**), *Dmt1* + *IRE* (**F**,**H**), *Dmt1*-*IRE* (**I**,**K**), and *Tfrc* (**J**,**L**) were measured in the renal medulla and cortex of WT, *Heph*/*Cp* KO, *Cp* KO, and *Heph* KO mice at 6 months of age by quantitative reverse-transcription polymerase chain reaction. *GAPDH* was used as a reference gene. Values are presented as mean ± SEM, *n* = 5 mice per group. Within each panel, bars without common letters are significantly different, *P* < 0.05.

*Cp, Heph, Fpn1, Dmt1* and *Tfrc* mRNA expression was examined by quantitative RT-PCR in the renal cortex and medulla of *Heph* KO*, Cp* KO, *Heph*/*Cp* KO and WT mice at 6 months of age (Fig. 4). There was no detectable *Cp* mRNA expression in *Heph*/*Cp* KO or *Cp* KO mice (Fig. 4A,C), and no *Heph* mRNA expression in *Heph*/*Cp* KO or *Heph* KO mouse kidneys (Fig. 4B,D). *Fpn1* and *Dmt1-IRE* mRNA expression levels were not significantly different between any of the mouse groups examined (Fig. 4E,G,I,K). *Dmt1* + *IRE* mRNA expression in the renal medulla, however, was significantly lower in *Heph*/*Cp* KO mice compared to WT mice (Fig. 4F), and *Dmt1* + *IRE* mRNA expression in the renal cortex was significantly higher in *Heph*/*Cp* KO mice compared to all other groups (Fig. 4H). *Tfrc* mRNA expression in the renal medulla was significantly lower in *Heph*/*Cp* KO and WT mice compared to *Heph* KO and *Cp* KO mice, and its expression in *Heph*/*Cp* KO medulla was significantly lower than that of WT mice (Fig. 4J). In contrast, *Tfrc* mRNA expression in the renal cortex was significantly higher in *Heph*/*Cp* KO mice compared to the other groups (Fig. 4L).

### Urinary iron, protein content and HE, PAS staining in the renal medulla and cortex

**Figure 5:**
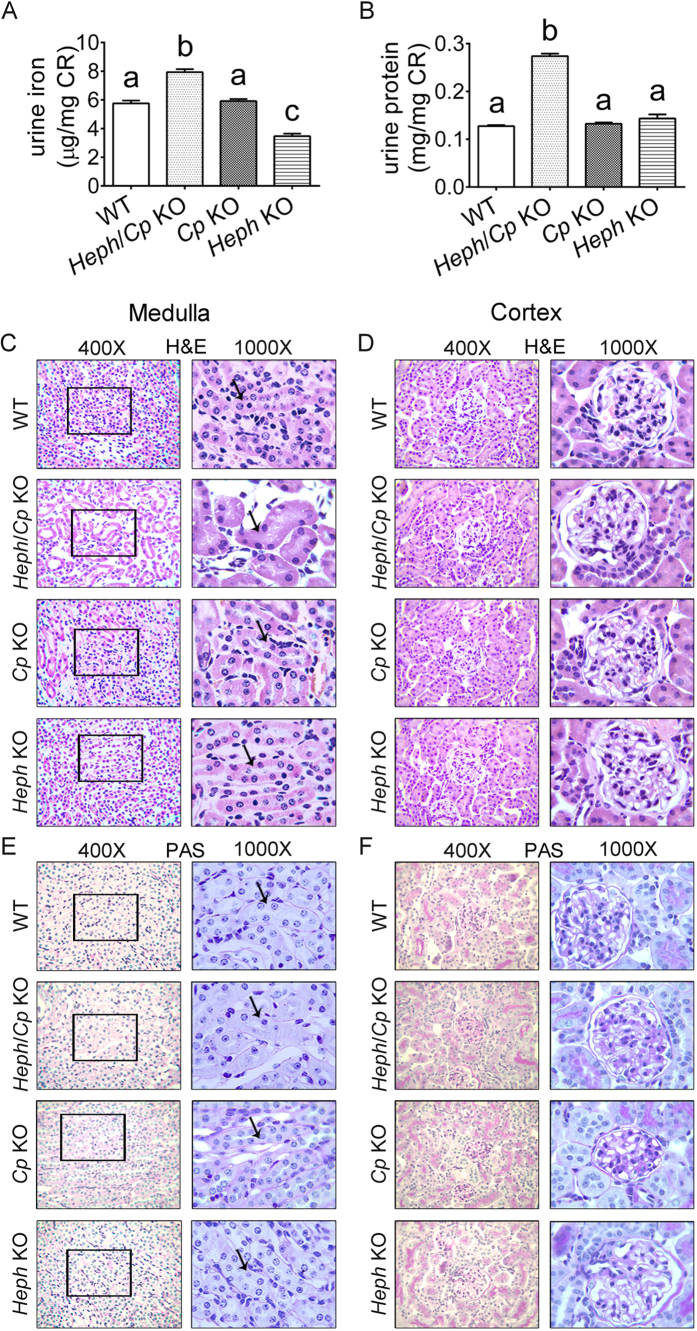
Urinary iron, protein content and H&E, PAS staining in the renal medulla and cortex. Total iron content in the urine of WT, *Heph*/*Cp* KO, *Cp* KO, and *Heph* KO mice was measured and total urine iron content was normalized to urine CR (**A**). Total protein concentration of WT, *Heph*/*Cp* KO, *Cp* KO, and *Heph* KO mice was measured and normalized to the urinary CR (**B**). Values are means ± SEM, n = 10 mice per group. Within each panel, bars without common letters are significantly different, P < 0.05. Representative photomicrographs of H&E and PAS staining for the tissues of renal medulla (**C**,**E**) and renal cortex (**D**,**F**) in WT, *Heph*/*Cp* KO, *Cp* KO, and *Heph* KO mice. The images were taken at high power (400×) and oil (1000×) lens.

Renal function was assessed by testing total urine iron and protein concentrations. Total iron content was measured in the urine of four group mice. Data were normalized to urine CR and expressed as microgram iron per milligram CR. The urinary iron and creatinine (CR) ratios were significantly increased in *Heph*/*Cp* KO mice compared to the other groups, and were significantly decreased in *Heph* KO mice compared to the other groups (Fig. 5A). The urinary protein and CR ratios were significantly increased in *Heph*/*Cp* KO mice compared to the other groups (Fig. 5B). The main renal injury location was in renal tubules in *Heph*/*Cp* KO mice. Morphological examination was performed by HE and PAS staining of renal medulla and cortex in four group mice, the results showed that renal tubular cells of medulla were dilated and nucleus shedding was obvious, and there was structural injury to the renal medulla in *Heph*/*Cp* KO mice. The number of renal tubular cells of medulla was lower in *Heph*/*Cp* KO mice compared to other three groups (Fig. 5C,E), but Morphological examination showed that there are no significant histological changes in the renal cortex of four group mice (Fig. 5D,F).

## Discussion

Our current study demonstrated that the iron concentration increased in the liver and decreased in the serum in *Cp* single and *Heph*/*Cp* double KO mice, and only increased in the kidney of *Heph*/*Cp* double KO mice. In *Heph* KO mice, however, there was a reduction in iron levels in plasma, liver and kidney (Table 2), as well as decreased urinary iron excretion. These data suggest that *Heph* KO mice have a systemic iron deficiency. *Heph*/*Cp* double KO mice had a low plasma iron content with increased total urinary iron, suggesting decreased renal iron reabsorption, which in turn probably induced renal injury. Previous research showed that mice with a combination of *Cp* loss and *sla* mutant *Heph* had high iron concentrations in the liver, brain, heart and pancreas^25^. The kidney was not examined. CP is mainly found as a soluble serum protein originating from the liver, but it is also found as a membrane-bound GPI-linked protein. The GIP-Cp first described to be present on the surface of astrocytes^26^, and current reported on the plasma membrane of spleen and kidney^6^. Our previous work demonstrated that HEPH is located in a supra-nuclear compartment and on the basolateral membrane of intestinal enterocytes^3^. Apical iron administration can lead to the mobilization of HEPH from intracellular sites to the basolateral membrane in MDCK cell line^4^. But the location of CP and HEPH in the kidney is still unclear. Our work shows that the deletion of either CP or HEPH has little impact on the kidney, but deletion of both MCFs leads to kidney iron deposition and this in turn impacts kidney function. These results suggest that there is redundancy in the role played by MCFs in renal iron homeostasis, with either single oxidase being able to sustain normal kidney iron metabolism when the other is lost.

CP and HEPH are MCFs that are known to work in concert with FPN1 to increase the efficiency of cellular iron export^27^. Existing data suggest that HEPH is the major oxidase involved in iron release from intestinal enterocytes^18^, while CP facilitates iron export from other tissues^8^,^19^. Our current work demonstrates that *Cp, Heph*, and *Fpn1* mRNA and protein are all present in the mouse kidney, consistent with previous studies^5^,^27^, and iron accumulation in the renal tubular cells and those in the Loop of Henle of *Heph*/*Cp* KO mice suggests that the oxidases are functional in this organ. Perls’ staining showed iron accumulation in both the renal medulla and cortex, but iron levels were highest in the medulla. The levels of ferritin protein expression in the medulla and cortex were consistent with elevated intracellular iron in these regions, and maximum iron accumulation in the medulla. The greater number of tubular cells (including distal tubular cells and those of the loop of Henle) in the renal medulla than in the renal cortex may explain this difference in iron load. Previous reports have suggested that most iron is reabsorbed by the proximal tubules, but the distal tubules also play a role in this process^12^. Our current data support the idea that the loop of Henle and the distal tubule also re-absorb iron. Compared with the renal medulla, the renal cortex had more glomerular and proximal tubular cells, the protein level of DMT1 was up-regulated in renal cortex of *Heph*/*Cp* KO mice may help to increase the iron transport and re-absorb when iron accumulated in the renal medulla of *Heph*/*Cp* KO mice, which may explain why the DMT1 protein was increased in the *Cp*/*Heph* KO mice cortex. Consistent with this, the cortex of the *Heph*/*Cp* KO mice had increased transferrin receptor and *Dmt1* mRNA levels.

This region is likely to be particularly important in conceiving iron that is not re-absorbed by the tubular cells of the cortex it induces part of cortex proximal tubular cells to up-regulated DMT1 protein levels to reabsorb urinary iron, which may explain why the DMT1 protein was increased in the *Cp*/*Heph* KO mice cortex. Consistent with this, the cortex of the *Heph*/*Cp* KO mice had increased transferrin receptor and *Dmt1* mRNA levels.

A small percentage of TF-bound iron is filtered through the glomeruli of the kidney^14^,^15^, and this iron is then reabsorbed by the proximal tubules^16^, Loop of Henle, and distal tubular cells^12^, likely still bound to TF. If these cells are unable to export iron across their basolateral membrane, intracellular iron accumulation would be expected. The apical supranuclear location of HEPH in MDCK and Caco2 cells, as well as results from colocalization studies in Cos7 cells, suggests that HEPH can localizes to recycling endosomes^4^. However, *in vivo*, in polarized mouse intestinal enterocytes, HEPH is present both on the lateral surfaces of enterocytes and also in the apical supranuclear region^3^, so precisely where it acts is unclear. If similar machinery is present in the kidney to enable vectorial transport of iron, relocalization of HEPH from a supranuclear compartment to the basolateral membrane may play a role in moving iron from the nephron lumen into the circulating blood, likely in conjunction with FPN1. *Fpn1* mRNA and protein levels in the kidney did not vary significantly between mice of different genotypes, suggesting that FPN1 protein levels are insensitive to kidney iron accumulation. Nevertheless, the efficiency of export can still be enhanced by MCFs.

Double knockout of *Heph* and *Cp* resulted in changes in the expression of other genes involved in iron metabolism. In the renal medulla, where ferritin protein expression and iron levels were significantly increased, *Dmt1* + *IRE* and *Tfrc* mRNA levels, and DMT1 and TFR1 protein expression were significantly reduced. Recent studies suggest that DMT1 is localized to the late endosomal and lysosomal membranes of proximal tubule cells, where it facilitates the uptake of TF-bound iron^16^,^28^. *Dmt1* + *IRE* and *Tfrc* mRNA levels can respond to the local iron status of the renal medulla, presumably through *IRP-IRE*–mediated mechanisms^29^,^30^,^31^. Wareing *et al*.^28^ have reported that rats fed an iron deficient diet had significantly increased renal DMT1 expression, while rats fed an iron-enriched diet showed a significant decrease in renal DMT1 expression. Thus renal DMT1 expression is sensitive to dietary iron content. *Dmt1* + *IRE* mRNA contains a single IRE in its 3′-untranslated region, similar to those in the *Tfrc* mRNA, and this element confers iron-dependent mRNA stabilization^30^. We hypothesize that lack of *Heph*/*Cp* results in reduced iron export from renal cells, which in turn results in cellular iron overload and decreased *Dmt1* and *Tfrc* expression.

We propose that the role played by HEPH or CP protein in the kidney is similar to that of other cells types. Under physiological conditions, iron reabsorption is mediated through the uptake diferric TF by TFR1 and/or cubilin on the apical surface of the epithelial cells in the renal tubules^10^,^15^. Transferrin-bound iron is transported into endosomes, where ferric iron dissociates from transferrin and is reduced to the ferrous state, possibly by STEAP3^32^,^33^. Iron is then exported from the endosomes into the cytoplasm by DMT1^16^. This iron can be utilized by cell itself, or exported back into the circulation via FPN1, a process in which HEPH and CP participate. Lack of HEPH and CP would be expected to block this final iron export step. Our current result has showed that iron reuptake is stuck in the apical side of renal tubular cells. It could be explained that a defective iron export at the basolateral membrane could block the mobilization of HEPH (maybe CP) from intracellular sites to the basolateral membrane, and cause stopping of iron transport at the apical side. For understanding a detailed process, the further study is needed.

Excess intracellular iron is toxic and prolonged exposure can lead to the generation of reactive oxygen species and tissue damage. Our demonstration that mice lacking both MCFs showed significant increase urinary iron excretion, proteinuria, as well as morphological abnormalities in the renal tubules suggests considerable structural and functional damage to the kidney. Together, these findings suggest that MCFs play an important role in protecting the kidney against injury.

In summary, our work has provided further insights into iron transport pathways in the kidney and has shown that copper-dependent ferroxidases play an important role in the homeostasis of this essential organ.

## Methods

### Mice

*Heph* KO and *Cp* KO mouse strains have been previously described^8^,^34^,^35^. The mice were originally obtained from the laboratory of Dr Greg Anderson at the QIMR Berghofer Medical Research Institute (Brisbane, Australia) and were bred and maintained at the Medical School of Nanjing University^36^. *Heph*^*−*/*−*^ (*Heph* KO) mice were bred to *Cp*^*−*/*−*^ (*Cp* KO) mice to generate *Heph*^*−*/*−*^/*Cp*^*−*/*−*^ (*Heph*/*Cp* KO) mice. All mice were on the C57BL/6J genetic background and were allowed unlimited access to a standard rodent unpurified diet containing approximately 180 mg/kg iron provided by Jiangsu Province Collaborative Medical Bioengineering Co., Ltd^37^. All animal studies were carried out in accordance with NIH guidelines, as described in the Guide for the Care and Use of Laboratory Animals of the NIH, and were approved by the Institutional Animal Care and Use Committee of Nanjing University.

### Tissue preparation

Male mice from each litter were euthanized at 6 months of age after anesthesia with an intra-peritoneal injection of 3% sodium pentobarbital. Blood was collected by cardiac puncture using potassium EDTA tubes (catalogue no. 367841, Becton Dickinson and Company, Franklin Lakes) and the liver and kidney were removed. Whole blood was used for hematological analysis and centrifuged to provide plasma for iron measurement as previously described^38^. A piece of liver and the whole left kidney were dried by heating at 80–100 °C for 2 hours and used for subsequent iron analysis. Four mice in each genotype group were perfused via the heart, first with phosphate buffered saline (PBS), then with 4% Paraformaldehyde (PFA). Collected tissues were immediately fixed in 4% PFA solution. Tissues were embedded in paraffin and sectioned for future immunostaining experiments. The kidney was dissected to separate the cortex and medulla. Samples of each were snap frozen in liquid nitrogen and then stored at −80 °C until required for RNA, protein, and iron concentration analyses.

### Tissue iron staining and iron status measurements

For immunostaining experiments, tissue was embedded in paraffin, sectioned and stained for ferric iron using Perls’ Prussian blue staining as previously described^35^. Other sections were detected with H&E or periodic acid Schiff (PAS) staining following the standard procedure. Blood hemoglobin and hematocrit levels were measured by an Automated Hematology Analyzer XS series (XS-800i Sysmex Corporation; Japan) in the Clinical Laboratory at Nanjing Drum Tower Hospital, The Affiliated Hospital of Nanjing University Medical School. The total concentration of iron in the dried tissues and plasma was measured using an Atomic Absorption Spectrometer (180–80, Hitachi; Japan) at the Modern Instrumental Analysis Center of Nanjing University after nitric acid digestion^38^. The concentration of non-heme iron in the renal cortex, renal medulla and total kidney was measured as previously described by a modification of the bathophenanthroline assay described by Torrance and Bothwell^39^. Data are expressed as μmol Fe/g dry weight or μmol Fe/L plasma.

### Total RNA extraction and qRT-PCR analysis

Total RNA was isolated from mouse tissues using TRIzol reagent (Invitrogen) according to the manufacturer’s protocol. Three micrograms of total RNA were reverse transcribed using a Transcriptor First stand cDNA Synthesis Kit (Roche Applied Science) and qRT-PCR was used to measure the expression of *Dmt1* (the transcripts with and without an iron responsive element; *Dmt1* + *IRE* and *Dmt1*-*IRE* respectively), *Fpn1, Heph, Cp*, and *Tfrc* in different parts of the kidney. All primers (Table 3) were designed by Primer3 software using the default settings^40^. Expression levels were normalized to that of the housekeeping gene *GAPDH*. Two-step PCR was monitored in real time by the FastStart Universal SYBR-Green Master (Roche Applied Science) according to the manufacturer’s instructions on an Applied Biosystems 7300 Real-Time PCR System instrument (Life Technologies, Shanghai, China). Fluorescence curves were analyzed with the LightCycler software (version 3.5.3). Automated calculation of crossing points was carried out by the second-derivative maximum method. Each reaction showed a single specific peak in the corresponding melting curve.

### Immunoblot analysis

Kidney regions from 6 month old WT, *Heph*/*Cp* KO, *Cp* KO, and *Heph* KO mice (n = 4–5 per genotype) were homogenized in 1 X PBS containing 1% Triton X-100, 0.1% SDS, and a protease inhibitor cocktail (Calbiochem.com, catalogue no. 539134). The total protein concentration was determined by the bicinchoninic acid (BCA) method (Bioworld Technology CO., MN). For most studies, samples containing 20–100 μg of protein were denatured by boiling for 5 minutes in 2 X SDS sample buffer. The proteins were separated by SDS–PAGE (7.5% acrylamide running gel) and transferred to nitrocellulose membranes. For FPN1 immunoblots, the samples were not heated. Blots were first incubated for 1 hour with blocking buffer (PBS containing 0.1% Tween-20 and 10% nonfat dry milk), and then incubated with primary antibodies overnight at 4 °C. The following primary antibodies were used: Anti-CP rabbit polyclonal antibody (1:1000; catalogue no. AP7340a, Abgent, CA); anti-HEPH rabbit polyclonal antibody raised against an N-terminus oligopeptide of hephaestin (Hp2, 1:1000)^4^; anti-FPN1 rabbit polyclonal antibody (Ireg1, 1:1000)^27^; anti-DMT1(IRE) rabbit antiserum (1:1000)^41^; anti-TFR1(CD71) mouse monoclonal antibody (1:1000; catalogue no. 136890, Life technologies, USA); anti-ferritin light chain (D-9) mouse monoclonal antibody (1:1000; catalogue no. sc-74513, Santa Cruz Biotech, CA); and anti-β-tubulin mouse monoclonal antibody (1:5000; catalogue no. M20005, Abmart, Shanghai, China). Blots were then washed 3 times in 0.1% PBS-Tween, incubated for 1 hour at room temperature with 1:2000 diluted peroxidase-labeled anti-mouse secondary antibody (catalogue no. sc-2031, Santa Cruz Biotech, CA) or 1:40000 diluted peroxidase-labeled anti-rabbit secondary antibody (catalogue no. sc-2030, Santa Cruz Biotech, CA), and signals were visualized by enhanced chemiluminescence (Thermo Scientific). Protein levels were then quantified by densitometry using UN-SCAN-IT software (Silk Scientific Corporation).

### Measurement of Urine iron, protein and Creatinine

Base-line urine was collected in all group mice. Total iron content in the urine of all group mice was measured using an atomic absorption spectrometer as previously described. Urinary protein concentration was measured using the bicinchoninic acid assay (Bioworld Technology CO., MN) and urine creatinine (CR) levels were measured using the creatinine assay kit (Jian Cheng Biological Engineering Institute, Nanjing, China) according to the manufacture’s instruction. Total urine iron content was normalized to urine CR and the urine protein excretion rate was expressed as the ratio of the urinary protein to urine CR.

### Statistical analysis

Unless otherwise indicated, values are presented as means ± SEM. One-way ANOVA with Tukey’s test for multiple comparisons was used to compare means for mouse tissue iron concentrations (i.e., liver, kidney, and plasma) and protein levels (i.e., L-ferritin, HEPH, CP, FPN1, DMT1 and TFR1) among the four groups. One-way ANOVA with Dunnett’s test for multiple comparisons was used to compare means for gene expression (i.e., *Heph, Cp, Fpn1, Dmt1* + *IRE, Dmt1*-*IRE*, and *Tfrc*) with WT control expression. Differences were considered statistically significant at P < 0.05. All statistical analyses were performed using GraphPad Prism 6 Software (GraphPad Software, San Diego, CA).

## Additional Information

**How to cite this article**: Jiang, B. *et al*. Hephaestin and ceruloplasmin facilitate iron metabolism in the mouse kidney. *Sci. Rep.* **6**, 39470; doi: 10.1038/srep39470 (2016).

**Publisher's note:** Springer Nature remains neutral with regard to jurisdictional claims in published maps and institutional affiliations.

## Figures and Tables

**Table 1:** Hematological parameters of MCF knockout and wild-type mice at 6 months of age.

Parameter	WT	*Heph*/*Cp* KO	*Cp* KO	*Heph* KO
RBC, X10^12^	7.44 ± 0.18^a^	2.92 ± 0.11^b^	7.56 ± 0.14^a^	5.71 ± 0.14^c^
Hb, g/L	108.8 ± 2.26^a^	45.2 ± 1.27^b^	103.2 ± 1.33^a^	79.6 ± 2.98^c^
Hct, %	34.7 ± 0.78^a^	10.8 ± 0.42^b^	33.7 ± 0.25^ac^	29.1 ± 0.47^c^
MCV, fL	46.7 ± 0.20^ac^	36.9 ± 0.15^b^	45.0 ± 1.07^a^	51.3 ± 0.68^c^
MCH, pg	14.5 ± 0.07^a^	15.7 ± 0.39^a^	13.7 ± 0.15^a^	13.8 ± 0.37^a^
MCHC, g/L	311 ± 1.02^a^	425 ± 14.7^b^	306 ± 4.74^a^	270 ± 8.97^a^
RDW, %	24.1 ± 0.25^a^	32.8 ± 0.31^b^	33.4 ± 0.33^b^	25.3 ± 0.98^a^

Mean ± SEM, n = 5–9. Results that do not share a letter are significantly different, P < 0.05. RBC, red blood cells; Hb, hemoglobin; Hct, hematocrit; MCV, mean corpuscular volume; MCH, mean corpuscular hemoglobin; MCHC, mean corpuscular hemoglobin concentration; RDW, red cell distribution width.

**Table 2:** Total iron and non-heme iron concentrations at 6 month of age.

Parameter	WT	*Heph*/*Cp* KO	*Cp* KO	*Heph* KO
Total iron				
Plasma, μmol/L	72.1 ± 0.30^a^	37.4 ± 0.35^b^	34.3 ± 1.00^b^	62.6 ± 1.45^c^
Liver, μmol/g dry weight	4.65 ± 0.11^a^	28.4 ± 1.44^b^	21.3 ± 0.49^b^	2.85 ± 0.06^a^
Kidney, μmol/g dry weight	3.85 ± 0.13^a^	12.1 ± 0.41^b^	3.41 ± 0.10^a^	2.79 ± 0.05^a^
Non-heme iron				
Kidney, μmol/g dry weight	3.32 ± 0.06^a^	11.7 ± 0.22^b^	3.21 ± 0.11^a^	2.68 ± 0.11^a^
Medulla, μmol/g dry weight	3.14 ± 0.13^a^	12.4 ± 0.27^b^	3.33 ± 0.13^a^	3.25 ± 0.02^a^
Cortex, μmol/g dry weight	3.35 ± 0.26^a^	11.1 ± 0.18^b^	3.29 ± 0.10^a^	2.81 ± 0.20^a^

Mean ± SEM, n = 7–10. Results that do not share a letter are significantly different, P < 0.05.

**Table 3:** Sequences of primers for quantitative RT-PCR.

Target	Forward primer	Reverse primer
*Cp*	TCTACCAAGGAGTAGCCAGGA	ATCTTCCCTCTCATCCGTGC
*Heph*	GAATTTTGCGAGCCGACCTT	TCATCCGCTTTCAGATACCC
*Fpn1*	TGTACCATGGATGGGTCCTT	TGCCACAACAACAATCCAGT
*Dmt1* + *IRE*	TAGGCTGTGCTCAAACCTACAGCA	TACATGAGAGCCAGGCATGGTAGA
*Dmt1-IRE*	CTCAGGTCTTCCTGGACAGC	CGCGTAGAGTGGGAAGAAAG
*Tfrc*	GGTGTTGCGGCGAAGTCCAGT	ACTCAGTGGCACCAACAGCTCC
*GAPDH*	AACTTTGGCATTGTGGAAGG	GGATGCAGGGATGATGTTCT

The targets are designated by their gene symbols.
